# Succinimidyl Alginate-Modified Fibrin Hydrogels from Human Plasma for Skin Tissue Engineering

**DOI:** 10.3390/gels11070540

**Published:** 2025-07-11

**Authors:** Ana Matesanz, Raúl Sanz-Horta, Alberto Gallardo, Cristina Quílez, Helmut Reinecke, Pablo Acedo, Diego Velasco, Enrique Martínez-Campos, José Luis Jorcano, Carlos Elvira

**Affiliations:** 1 Department of Bioengineering, Universidad Carlos III de Madrid (UC3M), 28911 Madrid, Spain; anuskamate@gmail.com (A.M.); cristina.quilez@uc3m.es (C.Q.); divelasc@ing.uc3m.es (D.V.); jjorcano@ing.uc3m.es (J.L.J.); 2 Department of Electronic Technology, Universidad Carlos III de Madrid (UC3M), 28911 Madrid, Spain; pag@ing.uc3m.es; 3 Department of Applied Macromolecular Chemistry, Institute of Polymer Science and Technology, Spanish National Research Council (ICTP-CSIC), Juan de la Cierva 3, 28006 Madrid, Spain; r.sanzhorta@gmail.com (R.S.-H.); gallardo@ictp.csic.es (A.G.); hreinecke@ictp.csic.es (H.R.); 4 Instituto de Investigación Sanitaria Gregorio Marañón, 28007 Madrid, Spain; 5 Group of Organic Synthesis and Bioevaluation, Instituto Pluridisciplinar, Universidad Complutense de Madrid (UCM), Associated Unit to the ICTP-IQM-CSIC, Paseo Juan XXIII, nº 1, 28040 Madrid, Spain

**Keywords:** fibrin hydrogels, plasma-derived fibrin hydrogel, succinimidyl alginate, tissue engineering, dermo-epidermal equivalents, in vitro human skin tissue engineering

## Abstract

Plasma-derived fibrin hydrogels are widely used in tissue engineering because of their excellent biological properties. Specifically, human plasma-derived fibrin hydrogels serve as 3D matrices for autologous skin graft production, skeletal muscle repair, and bone regeneration. Nevertheless, for advanced applications such as in vitro skin equivalents and engineered grafts, the intrinsic limitations of native fibrin hydrogels in terms of long-term mechanical stability and resistance to degradation need to be addressed to enhance the usefulness and application of these hydrogels in tissue engineering. In this study, we chemically modified plasma-derived fibrin by incorporating succinimidyl alginate (SA), a version of alginate chemically modified to introduce reactive succinimidyl groups. These NHS ester groups (N-hydroxysuccinimide esters), attached to the alginate backbone, are highly reactive toward the primary amine groups present in plasma proteins such as fibrinogen. When mixed with plasma, the NHS groups covalently bond to the amine groups in fibrin, forming stable amide linkages that reinforce the fibrin network during hydrogel formation. This chemical modification improved mechanical properties, reduces contraction, and enhanced the stability of the resulting hydrogels. Hydrogels were prepared with a final fibrinogen concentration of 1.2 mg/mL and SA concentrations of 0.5, 1, 2, and 3 mg/mL. The objective was to evaluate whether this modification could create a more stable matrix suitable for supporting skin tissue development. The mechanical and microstructure properties of these new hydrogels were evaluated, as were their biocompatibility and potential to create 3D skin models in vitro. Dermo-epidermal skin cultures with primary human fibroblast and keratinocyte cells on these matrices showed improved dermal stability and better tissue structure, particularly SA concentrations of 0.5 and 1 mg/mL, as confirmed by H&E (Hematoxylin and Eosin) staining and immunostaining assays. Overall, these results suggest that SA-functionalized fibrin hydrogels are promising candidates for creating more stable in vitro skin models and engineered skin grafts, as well as for other types of engineered tissues, potentially.

## 1. Introduction

Polymer functionalization plays a key role in modifying the properties of polymers for specific applications, such as hemostatic glue, drug and cell delivery carriers, tissue engineering matrices, hydrogels with cell viability, and other applications that have significant advances in bioengineering. Due to their excellent biological properties, hydrogels formed by natural polymers are frequently employed in bioengineering. However, these hydrogels suffer from some drawbacks, such as shrinking and degradation. Different approaches, including changes in composition and structure, have been developed to overcome these shortcomings and to improve the usefulness of these hydrogels. N-hydroxysuccinimide (NHS) is used in the field of natural polymers to either directly facilitate the formation of hydrogels using a bifunctional crosslinker, or to bond polymers, such as alginate [1], hyaluronic acid [2], chondroitin sulfate [3], etc., with other functional groups [4]. NHS is usually used in conjunction with a carbodiimide (EDC) that is in charge of activating the carboxylic acid, giving rise to the formation of a urea (unstable intermediate), which conjugates with NHS to give rise to the formation of an active ester in an aqueous medium that will form amides in the presence of primary amines [5]. EDC alone has been widely used to conjugate proteins and peptides or anchor other molecules of interest [6,7,8]. However, its use with NHS is recommended to improve the efficiency of the reactions because it has also been found that using these together is more productive when it comes to affecting the structure of the target proteins [9]. For example, NHS has been used in conjunction with the carbodiimide EDC to modify chondroitin sulfate or alginate in search of new materials with biomedical applications [10,11]. Numerous products can be prepared using this conjugation technique to obtain derivatives, such as hyaluronic acid, to obtain controlled protein release systems [12] or to encapsulate stem cells [13]. There is also wide use of these NHS/EDC coupling reactions for the surface immobilization of molecules of interest as biosensors [14]. Although these reactions are generally used directly, some authors have isolated natural polymers in which the carboxylic acid is activated by the NHS ester. Once modified, they use these NHS-activated polymers for the formation of pH-sensitive hydrogel [15], obtaining hydrogels based on hyaluronic acid functionalized with NHS ester in combination with free amine groups present on human blood proteins to develop cardiovascular therapies [16] or for the formation of adhesives for living tissues [17].

In this sense, hydrogels have emerged as an excellent material for tissue engineering due to their biocompatibility, high water content, and ability to mimic the extracellular matrix (ECM) of native tissues. In particular, in recent years, there has been an increasing demand in the development and production of in vitro-engineered skin substitutes that mimic human skin, either to be used as grafts to restore the damaged skin or for the establishment of human-based in vitro skin models for toxicity, cosmetic, and pharmaceutical testing [4,18,19,20]. This has led to the market revenues for bioengineered skin in vitro models reaching an approximate value of USD 4 billion in 2023, and those of the in vitro toxicology market are projected to reach USD 17.1 billion by 2028 [21].

Cellular bilayered constructs containing dermal and epidermal components have been widely used as they allow for the preparation of skin substitutes that better resemble the structure and function of human skin. The production of these constructs usually involves the use of different types of scaffolds composed of natural (such as alginate, collagen, chitosan, fibrin and hyaluronic acid) and/or synthetic (such as poly(ethylene glycol), poly-caprolactone, poly (vinyl alcohol), and poly(lactic acid)) polymers [22].

Natural materials, particularly collagen and fibrin, have been extensively used because they exhibit properties similar to those of the extracellular matrix of human tissues and organs, such as biocompatibility and mechanical performance [4,22]. Special attention has been paid to the use of human plasma-derived fibrin hydrogels as 3D scaffolds in skin engineering for clinical and experimental purposes [23]. Despite their frequent and fairly successful use, fibrin hydrogels have some drawbacks related to their poorly understood shrinkage, in-culture cell-promoted degradation, and poor mechanical properties, which make them difficult and mean that they need to be improved. Thus, a new experimental approach has recently emerged based on the development of improved fibrin scaffolds via fibrin modification and/or combination with other materials.

In this article, sodium alginate was modified using the EDC/NHS coupling reaction to obtain a succinimidyl alginate (SA). To chemically bond the amines in the plasma fibrinogen to the NHS ester in the SA solution to form SA-modified fibrin hydrogels (SA-fibrin), succinimidyl alginate was added to blood plasma before fibrin polymerization. The purpose of this process was to determine whether the changes in the structure and behavior of these new, modified hydrogels can allow us to overcome, at least partially, the drawbacks of unmodified plasma-derived fibrin hydrogels, described in the literature, and whether it can make the hydrogels more suitable as matrices for in vitro skin tissue culture [4]. We studied not only the synthesis and characterization of SA at different concentrations but also the consequences of its incorporation into plasma-derived fibrin hydrogels by studying aspects such as its gelation kinetics, microstructure, mechanical properties, and other important aspects such as the contraction of plasma-derived fibrin/SA hydrogels, their lack of toxicity in primary human fibroblast (hFBs) and keratinocyte (hKCs) cell cultures, and their capacity to support the in vitro formation of 3D dermo-epidermal skin substitutes.

## 2. Results and Discussion

### 2.1. Synthesis and Characterization of Succinimidyl Alginate (SA)

Succinimidyl alginate (SA) was synthesized using different molar ratios (1:1:1, 1:1:3 and 3:1:3) of Alginate:EDC:NHS to analyze the amount of NHS ester present in the polymer. Furthermore, we used different solvents for the precipitation of SA, analyzing whether they had any effect on the amount of NHS ester obtained. To ensure that SA had the optimal degree of modification, 26% of NHS ester was used, and the conditions mentioned in the Materials and Methods section were chosen, these including a degree of functionalization similar to that used in other publications [15]. Upon precipitation and drying, a fibrous material with a whitish appearance and a slight yellowish tone was obtained. As can be seen in Table 1, the difference in modification depends on the reaction time and the solvent used to precipitate the resulting product. Isopropanol was the chosen solvent because it was easier to remove by vacuum drying, since others that were assayed appeared as impurities in the SA ^1^H-NMR spectrum.

**Figure 1:**
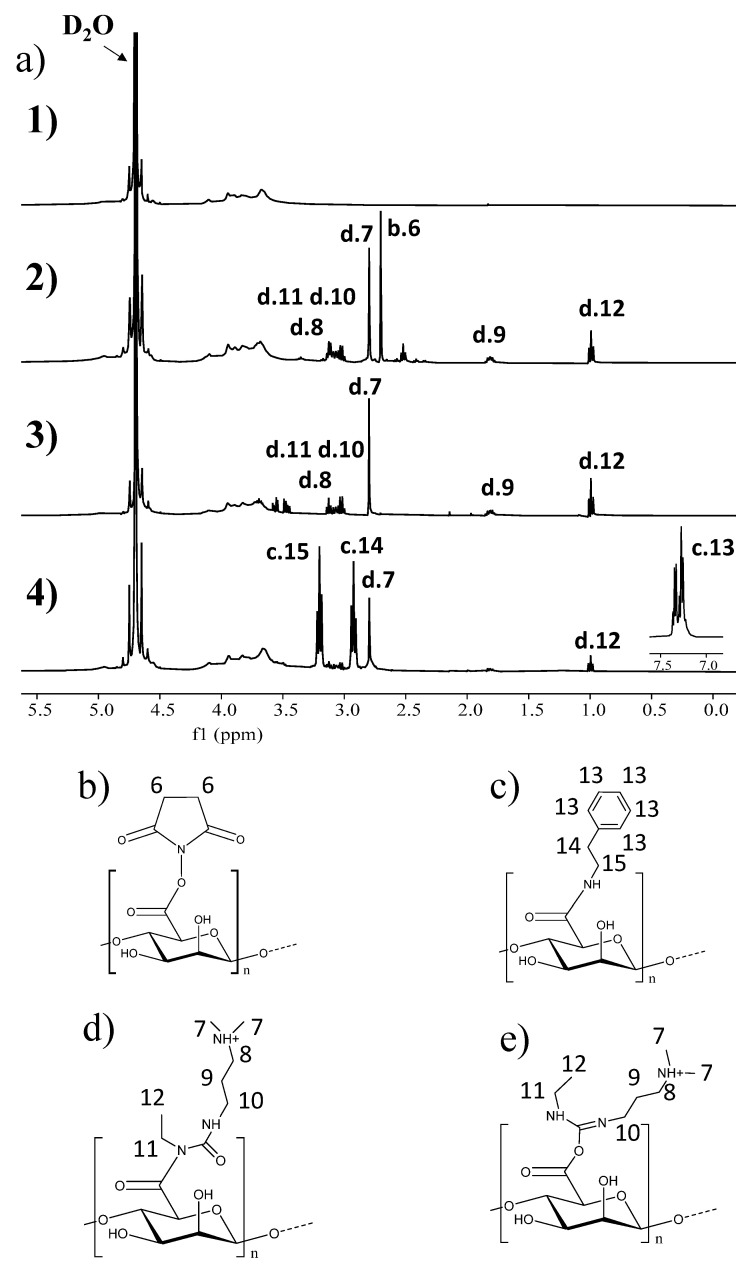
(**a**) 1H-NMR spectra of (**a1**) sodium alginate, (**a2**) succinimidyl alginate after reaction with EDC:NHS, including structures (**b**,**d**,**e**), (**a3**) succinimidyl alginate after 7 days of dialysis in water including structures (**b**,**d**,**e**) and (**a4**) succinimidyl alginate after reaction with phenylethylamine, including the structure of (**c**) after reaction with phenylethylamine. Structures of (**b**) succinimidyl alginate, (**c**) phenylethylamine attached to the alginate, (**d**) N-acylurea attached to the alginate and (**e**) the intermediate O-acylurea complexed with the alginate.

Analyzing Figure 1, in the ^1^H-NMR spectrum of SA compared to sodium alginate, two very pronounced signals are observed at 2.7 and 2.8 ppm [24,25]. The signal at 2.7 ppm corresponds to the methyl groups of the succinimidyl ester unit (4H, m, 2CH_2_ NHS). The signal at 2.8 ppm corresponds to the methyl groups of the carbodiimide (6H, m, 2CH_3_, EDC and EDU) that also coincides with the methyl groups of the urea EDU (N-ethyl-N’-(dimethylaminopropyl) hydrolyzed carbodiimide and its respective urea derivative), resulting from the reaction [26,27]. The ^1^H-NMR spectrum of the control alginate was obtained by pre-saturation and at a temperature of 80 °C to shift the signals to avoid thse overlapping with the signal of the deuterated solvent, in this case water. In this sense, the starting alginate had the following proportions of mannuronic and guluronic acid: 0.54:0.46. Thus, in Figure 1(a2), the signals belonging to alginate and succinimidyl alginate can already be associated with each other, obtaining a 26% modification. Using GPC, the molecular weight of SA was determined, giving a number average molecular weight (Mn) of 222 kDa and a polydispersity index (PI) of 2.26, while for the control alginate, an Mn of 200 kDa was obtained, with an IP of 2.32. This slight increase in molecular weight after the reaction may be due to the formation of crosslinking sites due to EDC, causing reactions between the carboxyl group and different hydroxyl moieties present in the alginate repeating unit [28]. For this reason, an excess of NHS was used in order to prevent this type of crosslinking.

To confirm that the NHS ester is capable of reacting with amine products and to observe if the percentage of modification corresponds to that measured by NMR, the SA was reacted with phenylethylamine in an aqueous medium, with an excess of amines added in a ratio of 3:1 with respect to the amount of NHS ester. The solution was left to dialyze for several days to eliminate the excess of unanchored phenylethylamine, and then the resulting product, which can be observed in Figure 1(a4), was lyophilized. In the ^1^H-NMR spectrum, the presence of the characteristic aromatic ring of phenylethylamine between 7 and 7.5 ppm can be observed, and it can also help us to verify the degree of modification achieved, coinciding with the value of 28% estimated before. In this sense, it can be confirmed that succinimidyl alginate is capable of anchoring or reacting with amino products, as other authors have previously verified [29]. In addition, in this spectrum, the characteristic signals of N-acylurea (Figure 1(a1,a3),d,e) can be observed; it does not react with primary amines, but neither does it prevent the anchoring of other amine products.

**Figure 2:**
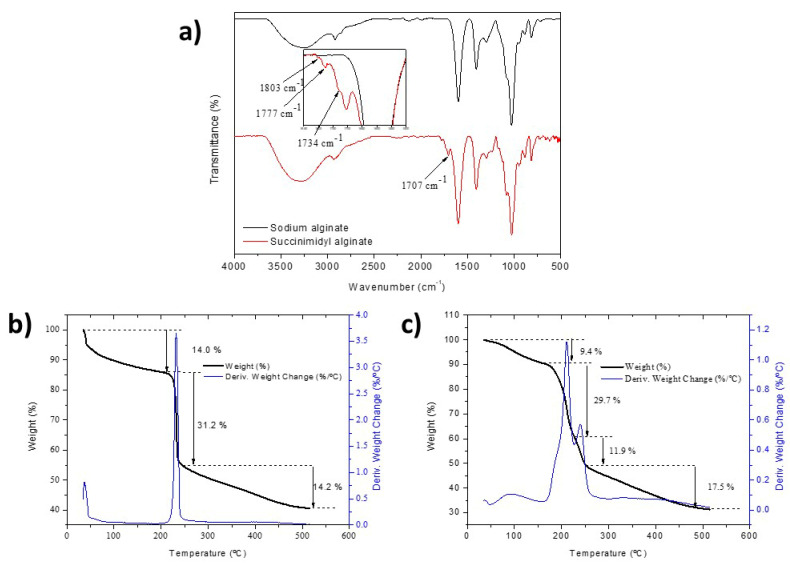
(**a**) FTIR-ATR spectra of sodium alginate and succinimidyl alginate. (**b**) TGA thermograms of sodium alginate and (**c**) succinimidyl alginate.

To complete characterization, ATR-FTIR spectra of the control sodium alginate and SA were compared, and a thermogravimetric analysis to study the formation of NHS ester and N-acylurea was carried out. Four new significant signals can be distinguished in the region between 1650 and 1825 cm^−1^ in the infrared spectra of Figure 2a. The signals at 1734, 1777 and 1803 cm^−1^ correspond to the formation of the NHS ester. On the other hand, a pronounced signal at 1700 cm^−1^ corresponding to the stretching vibration of the C=O carbonyl of N-acylurea can observed, and this has also been detected in other studies [30,31]. The thermogravimetric analysis of Figure 2b shows that, in the region previously associated with the decomposition of the carboxylic acid, there is another instance of weight loss that can be associated with the decomposition of either the succinimidyl ester, the N-acylurea or both of them at the same time. Using the percentages of weight loss, it is possible to estimate the degree of modification, although a more exhaustive study would have to be carried out since the signals corresponding to the weight loss of NHS ester and N-acylurea overlap, so we maintain the percentages of functionalization obtained by ^1^H-NMR.

### 2.2. Incorporation of SA into Human Plasma-Derived Fibrin Hydrogels

Once obtained and characterized, SA was used to generate modified plasma-derived fibrin hydrogels. The goal was to produce hydrogels with a final fibrin concentration of 1.2 mg/mL, as described in Section 4.2.6.

First, we examined whether the addition of SA had any inhibitory effect on polymerization or delayed the gelation time of the hydrogels, as was previously reported when other macromolecules were used.

**Figure 3:**
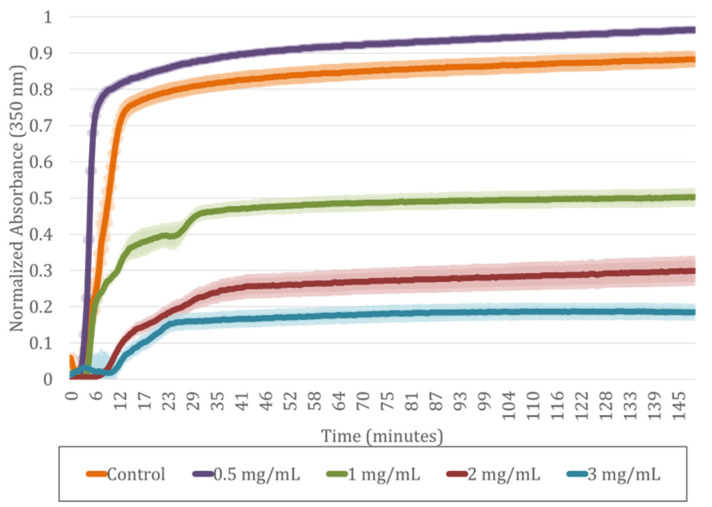
Polymerization kinetics for plasma-derived fibrin hydrogels modified with different SA concentrations (mg/mL) measured by normalized absorbance at 350 nm. Data expressed as mean ± standard deviation and N = 3.

The flip-flop assay demonstrated that increasing SA concentrations caused a moderate delay in gelation but did not inhibit fibrin polymerization. As shown in Table 2, the highest concentration of SA in the hydrogels without compromising gelation was 4 mg/mL. The control (0 mg/mL SA) polymerized in 15 min, while at 0.5 mg/mL, gelation occurred slightly faster (10 min). From 1 to 4 mg/mL SA, a gradual delay in the polymerization time was observed, this reaching up to 20 min at the highest concentrations. Thus, the presence of SA slightly slows down gelation, but it does not prevent hydrogel formation, confirming that polymerization is always completed. This delay is also consistent with the behavior observed in the UV spectroscopy analysis shown in Figure 3.

For studying how SA incorporation affects plasma-derived fibrin hydrogel formation and polymerization kinetics, the absorbance of the polymerizing solutions was measured using UV spectroscopy after CaCl_2_ addition (see Figure 3). We observed, have the authors of other published studies [2,32,33,34,35,36,37,38,39], that as the SA concentration increased, the hydrogels became more transparent, as confirmed by a decrease of up to 85% in absorbance at the plateau compared to the control. This higher transparency is attributed to the increased crosslinking density caused by the reaction of NHS esters with primary amines, resulting in a denser and more uniform network that reduces light scattering.

No clear effect of the inhibition of fibrin polymerization was observed. However, small steps in the absorbance curves between 1 and 3 mg/mL SA suggest a gradual stabilization of the network after initial gelation. This behavior likely results from the interaction between CaCl_2_ and carboxylic groups in SA, which may transiently chelate Ca^2+^ ions to form physical hydrogels and a dual competition for Ca^2+^ in fibrin [40] and SA [41] that slightly delays polymerization. In the case of the hydrogel with an SA concentration of 0.5 mg/mL, a slight increase in the turbidity of the hydrogel was observed, the correlation of which with the microstructure will be discussed later; furthermore, quicker polymerization was observed.

Therefore, both the flip-flop assay (see Table 2) and UV spectroscopy (see Figure 3) indicate that increasing the SA concentration causes a moderate delay in polymerization (from 10 to 20 min), without inhibiting it, and leads to the formation of more transparent hydrogels.

### 2.3. Microstructure of SA-Modified Plasma-Derived Fibrin Hydrogels

**Figure 4:**
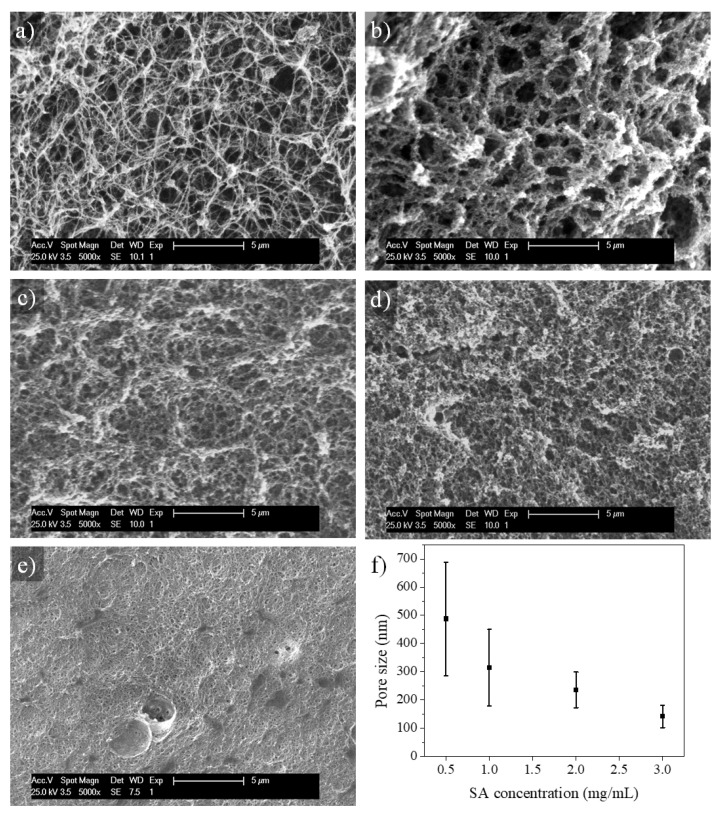
SEM micrographs showing the microstructure of plasma-derived fibrin hydrogels modified with different concentrations of SA: (**a**) control, (**b**) 0.5 mg/mL, (**c**) 1 mg/mL, (**d**) 2 mg/mL and (**e**) 3 mg/mL. (**f**) Graph of the pore sizes observed in the microstructure of the hydrogels versus the SA concentrations. Data expressed as mean ± standard deviation. N = 3.

The effect of SA on the microstructure of the plasma-derived fibrin hydrogels was evaluated by SEM analysis (Figure 4). The plasma-derived fibrin hydrogels modified with different concentrations of SA were dried by processing them in supercritical CO_2_. In Figure 4a, it can be observed that the control hydrogel displays the filamentous microstructure characteristic of plasma-derived fibrin hydrogels (see Figure 4a) [42]. Upon SA addition, it was observed that this microstructure progressively changed into a microporous one (Figure 4b–e). Furthermore, pore size decreased as SA concentration increased. At an SA concentration of 0.5 mg/mL, the initial formation of fibers was observed, and these finally aggregated, giving rise to a porous morphology. The pores observed were similar in size to those of the control hydrogel, with the difference being that their component fibers were thicker and more aggregated. As a consequence, their porosity was lower than that of control hydrogels. In the most extreme case, for an SA concentration of 3 mg/mL, the microstructure of the hydrogels was porous and very dense (Figure 4e). These structural modifications align with the enhanced optical transparency observed in the UV absorbance assays we conducted (see Section 2.2), as denser and more homogenous fiber networks reduce light scattering. The increase in transparency is therefore consistent with the more compact microarchitecture seen in SEM images. This hydrogel microstructure is a result of the activity of succinimidyl esters [35], which tend to cause fiber aggregation during fibrin polymerization, and partially the result of the ability of alginate to form polymer hydrogels in the presence of divalent ions, in this case Ca^2+^, which interfere with fibrin polymerization. Figure 4f shows the above-mentioned effect of SA concentration on the size of pores formed in fibrin hydrogels, which decreases with increasing SA concentration.

The lower-porosity, smaller-pore-size, and thicker fibers observed at higher SA concentrations could be harmful for cell growth. In addition, as we will show later, higher concentrations of SA improve the mechanical properties of fibrin hydrogels. Therefore, it is necessary to carry out cell viability studies in 2D and 3D cultures, as we will also show below, to optimize the level of SA modification in SA–fibrin hydrogels.

### 2.4. Long-Term Stability of SA-Modified Plasma-Derived Fibrin Hydrogels

**Figure 5:**
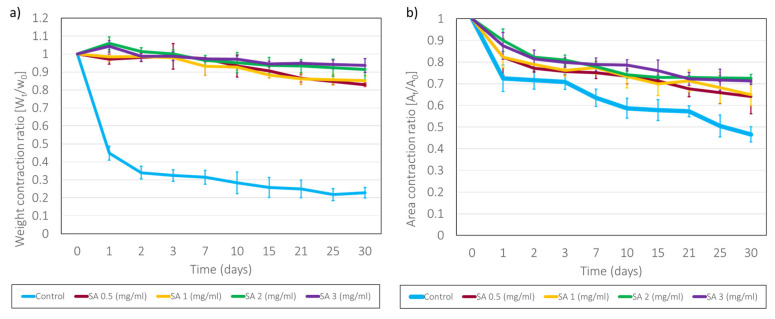
(**a**) Graph showing the contraction kinetics of plasma-derived fibrin hydrogels, as a function of weight, modified with different concentrations of SA. (**b**) Deswelling kinetics of plasma-derived fibrin hydrogels, as a function of area, modified with different concentrations of SA. Data are expressed as mean ± standard deviation N = 3–6.

Through a study of the evolution of the weight of the hydrogels submerged in PBS, their long-term stability in aqueous medium was verified. As can be observed in Figure 5, the SA-modified hydrogels shrank by approximately 10–25% in weight after 30 days of being immersed in PBS, unlike the control fibrin hydrogels (derived from plasma), which shrank by up to 80% (70% in the first 24 h). It was observed that during the first two days, contractions were relatively rapid, followed by slow contractions over longer periods, while the control hydrogel stabilized its weight loss after 24 h. It is also worth noting that the kinetics of SA 0.5 and 1.0 are very similar, whereas that of 2.0, despite having the same shape, exhibits statistically significantly higher shrinkage. In other cases, NHS cross-linking has been observed to improve the stability and degree of deswelling of hydrogels [16,31]. This is due to the experimental process, since the hydrogels were made in glass vials, from which they were later detached by adding PBS. While plasma-derived fibrin hydrogels detached easily, as do slightly modified ones, hydrogels with a concentration of SA of 2 mg/mL adhered strongly to glass and were very difficult to detach from the vials, sometimes breaking during peeling. An attempt was made to carry out this assay with the 3 mg/mL hydrogels, but they adhered so much to the vials that it was impossible to peel them off without breaking them. This ability to adhere, although detrimental to the development of this assay, presents an advantage in terms of the development of dermo-epidermal cultures, which require that the hydrogel remains perfectly adhered to the substrate where it has been gelled. In this regard, many authors have considered it appropriate to modify polymers using an NHS ester so that these materials adhere to living tissues for different applications in biomedicine [2,36].

When measuring the area of the hydrogels, as can be seen in Figure 5, the control fibrin hydrogels showed a reduction in surface area of approximately 45%, while for the hydrogels modified with different concentrations, this surface contraction reduced to approximately 25% after 30 days for the three SA concentrations analyzed. The differences in results between area and weight contraction studies may be caused by the fact that the area ratios did not include a consideration of contraction in Z, but only in X and Y, as well as the fact that the areas were measured in culture media, so the hydrogels may have been swelling, which was not the case when measuring the weight ratio. The hydrogels modified with a concentration of 2 mg/mL of SA seem to have maintained their surface contraction without breaking.

The ability for the hydrogels to remain stable in an aqueous medium may have been due to the hydrophilicity that the alginate itself possessed, which would have provided the hydrogels with a greater ability to swell in the aqueous medium. On the other hand, this may also be associated with the previously observed change in the microstructure, where the obtained micropores may have helped to keep the hydrogel structure stable upon swelling and may also have allowed for greater swelling capacity; the latter might also be the reason why the modified hydrogels were capable of reducing surface shrinkage [43]. On day 30 of the test, it was observed that the control fibrin hydrogels were very cloudy and had weak mechanical properties, while the modified ones appeared more transparent and more resistant. All these changes in properties were caused by the formation of amides between the succinimidyl ester, present in SA, and the primary amines, present in various plasma proteins, including fibrinogen, which gave rise to more cross-linked systems that resulted in the greater stability of the hydrogels [44].

### 2.5. Stability of SA-Modified Plasma-Derived Fibrin Hydrogels Containing hFBs

**Figure 6:**
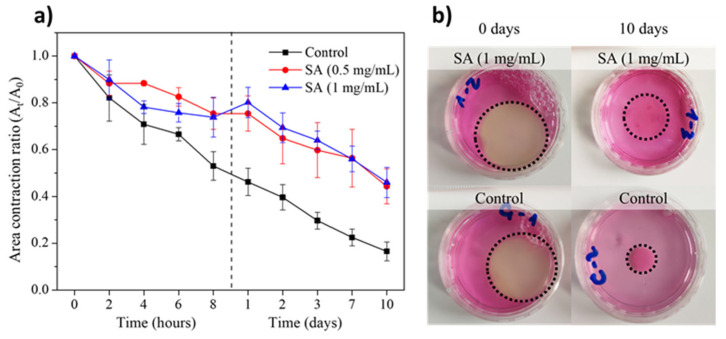
(**a**) Deswelling kinetics of plasma-derived fibrin hydrogels containing hFBs without and with SA at concentrations of 0.5 and 1 mg/mL measured through differences in area. (**b**) Images of fibrin hydrogels containing hFBs, without (**lower**) and with (**upper**) SA in an amount of 1 mg/mL, at day 0 (**left**) and day 10 (**right**). Hydrogels in (**a**,**b**) were kept submerged in culture media on Petri dishes P60. The initial hydrogel area was 7.07 cm^2^. Dotted lines represent the hydrogel’s limits in the Petri dishes. Data are expressed as mean ± standard deviation, N = 3.

The surface contraction in plasma-derived fibrin hydrogels with embedded hFBs was also evaluated. The cells were incorporated into the hydrogels just before the addition of CaCl_2_ to induce fibrin polymerization. As shown in Figure 6a, the surface area of the cell-integrated SA-free control hydrogels decreased linearly over time to a value of 25% by day 10.

On the other hand, the cell-integrated SA-containing hydrogels were observed to have a surface area that reduced significantly more slowly, reaching a value of 50% of the initial area at 10 days and with no statistically significant differences at the SA concentrations studied. These results confirm that the incorporation of SA in fibrin hydrogels improves their stability even under cell culture conditions. A comparison of Figure 5 and Figure 6 shows that the presence of hFBs leads to stronger contraction of the gels, which is expected given the existing published results on the ability of fibroblasts to contract and degrade fibrin and collagen hydrogels [4,42,45,46,47,48,49].

The above results are shown in a visual form in Figure 6b: hydrogels shrink in the presence of hFBs, and the area contraction of hydrogels containing SA is smaller than that of control fibrin hydrogels. On day 0, the hydrogels containing 1 mg/mL of SA and the control hydrogels show a visual similarity that represents the hydrogels without contractions. However, when compared at day 10, the SA-modified hydrogel showed a contraction of approximately 50% of its initial area, while the control one showed much larger contraction, this reaching 15% of its initial area. Therefore, hydrogels containing hFBs behave differently to those without cells (Figure 5), demonstrating that, although there is a contribution of SA to reducing contraction in both cases, the presence of hFBs provokes additional contraction due to the presence of fibroblasts.

### 2.6. Rheological Properties of SA-Modified Plasma-Derived Fibrin Hydrogels

**Figure 7:**
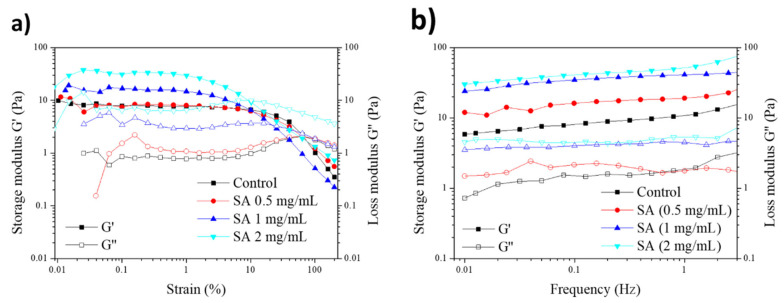
Rheological properties of the hydrogels modified at different concentrations of SA, measured by the following tests: (**a**) strain scanning and (**b**) frequency scanning.

The mechanical behavior of hydrogels modified by SA was studied through rheological tests. First, the region of linear viscoelasticity of each modified hydrogel was analyzed for variations, using a strain scanning test with a strain between 0.01% and 200%. In this test, and as can be observed in Figure 7, when the hydrogels reached a certain percentage of deformation, the values of G′ and G″ intersected; initially showing solid behavior, they changed to show to liquid-like behavior. This defines the zone of linear viscoelasticity in which G′ and G″ remain constant, the conditions being G′ > G″ for a given range of deformations. This is important because it reflects the mechanical behavior of each structure. The fibrin control gel, the mechanical behavior of which is defined by the fibers, is elastic but less resistant. On the other hand, the modified gel is porous and denser, resulting in a higher elastic modulus but faster plastic deformation due to its microporous structure. Following this test, it could be observed that G″ evolved in such a way that, for high deformations, it reached a maximum value characteristic of the energy dissipation that occurred when the hydrogel breaks its structure. Figure 7a shows how, when increasing the concentration of SA in the hydrogel, the value of G′ increases, leading to the obtention of more elastic and rigid hydrogels in comparison to the control fibrin hydrogels. These results support the SEM data, which indicated that hydrogels containing SA exhibited a more compact microstructure with smaller pores and less porosity as the concentration increased. Additionally, they showed reduced weight and area shrinkage, leading to hydrogels that were stiffer and had higher G′ values. However, it was observed that the G″ peak that delimited the transition from solid to liquid behavior was found at lower deformations, reducing the range of linear viscoelasticity of the modified hydrogels.

Once the region of linear viscoelasticity for each one of the hydrogels modified with SA was known, a frequency sweep test was carried out for a given percentage of deformation, which in this case was 1% since this falls within the range of linear viscoelasticity in all cases. In this case, it was observed that all the hydrogels maintained a G′ value above the G″ value and that this remained constant for the frequency sweep. The storage modulus values increased as the concentration of SA in the hydrogel was increased, aligning with the changes previously seen in the microstructure, which in turn generated a change in the hydrogel’s mechanical properties and viscoelastic behavior. The viscoelastic behavior of plasma-derived fibrin hydrogels is defined by their micro-fibrous microstructure, which determines the elasticity and energy dissipation capacity of the system. However, in the case of the modified hydrogels we studied, their microporous structure seemed to reduce the region of linear viscoelasticity, although this did make the hydrogel present a more resistant elastic part compared to unmodified hydrogels.

### 2.7. Viability and Proliferation of Primary Human Fibroblasts (hFBs) and Immortalized Human Keratinocytes (HaCaT) in Plasma-Derived Fibrin/SA Hydrogels

**Figure 8:**
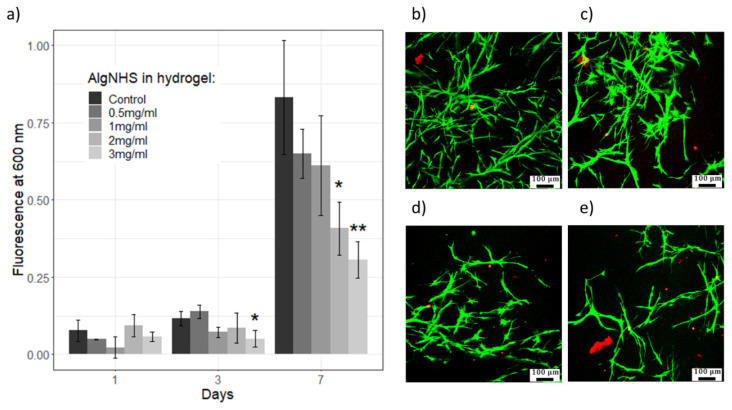
AlamarBlue (**a**) and Live/Dead assays (**b**–**e**) of hFBs embedded in control (**b**) and SA-modified fibrin hydrogels (**c**–**e**). (**a**) Normalized proliferation measured by the AlamarBlue™ assay at 1, 3 and 7 days of cell culture. Fluorescence was measured at excitation/emission wavelengths of 530 nm/600 nm. Data expressed as mean ± standard deviation (N = 3) (* *p* < 0.05, ** *p* < 0.01). The *Y* axis represents normalized fluorescence intensity, reflecting the relative metabolic activity of the cells. (**b**–**e**) Images obtained by confocal microscopy of hydrogels at 7 days of culture of (**b**) the control plasma-derived fibrin hydrogel, (**c**) the hydrogel modified with 0.5 mg/mL of SA, (**d**) the hydrogel modified with 1 mg/mL of SA, and (**e**) the hydrogel modified with 2 mg/mL of SA. Scale bar 100 µm.

Using the AlamarBlue assay, the proliferation of human fibroblasts (hFBs) embedded in SA-modified plasma-derived fibrin hydrogels was studied at different concentrations of succinimidyl-alginate (SA) (0.5, 1, 2, and 3 mg/mL) for one week. The results showed a significant increase in hFB proliferation between days 3 and 7 for all SA concentrations (see Figure 8a), consistent with previously published results on the effect of combined alginate and plasma-derived fibrin hydrogels on cell proliferation [50,51]. Images show that hFBs remained attached and maintained their morphology regardless of SA polymer addition, suggesting that key parameters such as cell attachment and spreading were not substantially affected (see Figure 8b–e). However, the highest SA concentrations (2 and 3 mg/mL) inhibited hFB proliferation in the modified hydrogels compared to that in the control hydrogels, in a statistically significant manner (* *p* < 0.05). This effect may be attributed to several factors, including the microstructure of the hydrogels. As shown in Figure 3, as the SA concentration increased, the internal microstructure of the hydrogels became denser and less porous, impairing cell proliferation and migration, and limiting the diffusion of nutrients. Additionally, the NHS ester groups of modified alginate may have interfered with the RGD motifs present in fibrin, which are crucial for cellular adhesion and proliferation. These findings align with previous work by Wang et al., who reported that chemical modifications of alginate with the NHS matrix can affect cell–hydrogel interactions by altering the accessibility of adhesion motifs like RGD, thereby impairing cellular adhesion and proliferation [36,52,53].

At day 7, SA concentrations of 0.5 and 1 mg/mL supported cell proliferation at levels statistically similar to those of the plasma-derived fibrin hydrogels, whereas higher concentrations of SA led to reduced cell growth. Additionally, to ensure that the hydrogel material itself did not interfere with assay readings, AlamarBlue assays were conducted with acellular hydrogels. No significant differences were observed between hydrogels with different SA concentrations in the absence of cells, confirming the reliability of the AlamarBlue data for subsequent cell studies. These results demonstrate the need to fine-tune the SA concentration to optimize the relationship between the improved mechanical properties of hydrogels and cell proliferation.

In addition, to verify whether the effect of SA on hFB proliferation was, at least in part, cytotoxic in nature, Live/Dead assays were performed at seven days of culture (Figure 8b–e). A progressive decrease in the number of hFBs was observed with increasing SA concentrations, corroborating the results obtained with AlamarBlue. The fact that almost all cells fluoresced green, i.e., live cells in this assay, together with the virtual absence of cells fluorescing red, i.e., dead cells in this assay, indicates that the observed cell number decreased, which indeed seems to be due to the lower proliferation of fibroblasts. Finally, with increases in the SA concentration in the hydrogels, the fibroblasts appeared to have developed a less elongated and filamentous appearance, which aligns with the higher stiffness and lower porosity (lower pore number and size) observed in these hydrogels (see Figure 4 and Figure 7). This change in morphology further indicates the impact of the mechanical properties of a hydrogel on cellular behavior.

**Figure 9:**
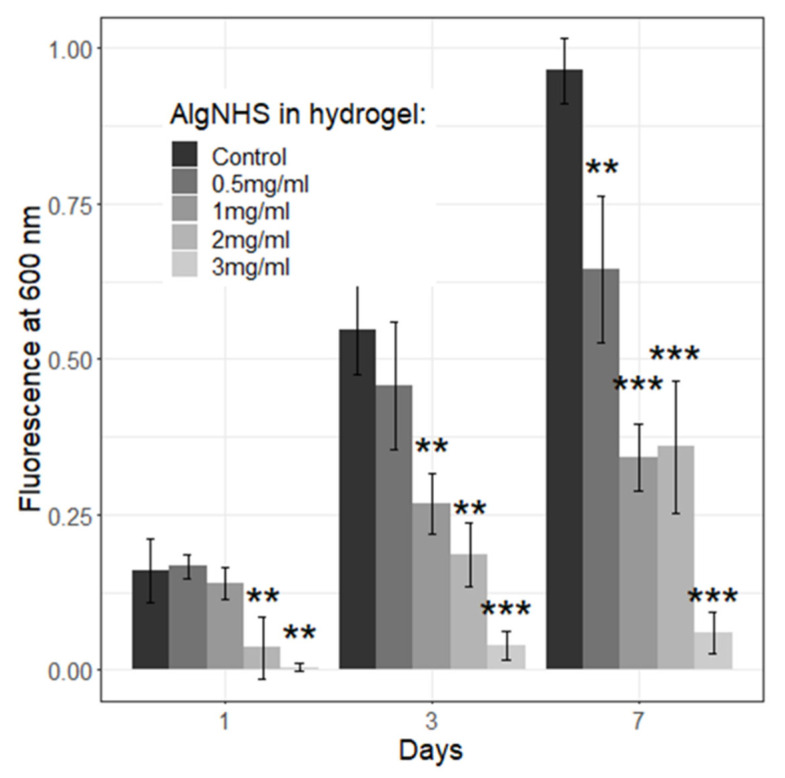
AlamarBlue assays of HaCaT cells seeded on top of SA–plasma-derived fibrin hydrogels. Normalized proliferation was measured by the AlamarBlue™ assay at 1, 3 and 7 days of cell culture. Fluorescence was measured at excitation/emission wavelengths of 530 nm/600 nm. Data are expressed as mean ± standard deviation (N = 3) (** *p* < 0.01, *** *p* < 0.001). The *Y* axis represents the normalized fluorescence intensity, reflecting the relative metabolic activity of the cells.

To model the epidermal compartment, similar experiments were performed with HaCaT keratinocytes seeded on top of the hydrogels. The results showed a similar trend (see Figure 9), with HaCaT cell proliferation decreasing progressively with increasing SA concentrations, particularly at concentrations above 1 mg/mL, as well as in the control samples. At these higher concentrations, the negative effects on keratinocyte proliferation were more pronounced. These findings suggest that, for optimal cellular biocompatibility, SA concentrations should not exceed 1 mg/mL in future experiments. This decrease in proliferation can be attributed to mechanisms similar to those affecting fibroblasts, as described above. This is consistent with previous reports that emphasize the importance of maintaining a balance between matrix mechanical properties and cell adhesion sites [54,55,56].

In conclusion, the viability and proliferation studies demonstrated that low concentrations of SA (0.5–1 mg/mL) in fibrin hydrogels provide appropriate biocompatibility for both dermal fibroblasts and epidermal keratinocytes and increase hydrogels’ mechanical properties, indicating that these modified hydrogels constitute interesting new materials in the development of dermo-epidermal skin equivalents.

### 2.8. Plasma-Derived Fibrin/SA Dermo-Epidermal Skin Equivalents Containing Human Primary Cells

Altogether, the results so far presented indicate that, in terms of reduced contraction, viscoelastic moduli (G′, G″), and cell viability and proliferation, fibrin gels with SA concentrations in the range of 0.5–1 mg/mL represent interesting new materials for the production of dermo-epidermal equivalents. However, for these matrices to be considered suitable for the production of skin equivalents for experimental, clinical or drug testing purposes, they must allow for the development of bi-layered skins (with dermis and epidermis) that histologically well-organized and have a well-differentiated epidermis.

Dermo-epidermal skin equivalents were developed in hydrogels containing 1.2 mg/mL of plasma-derived fibrin and 0.5 or mg/mL of SA as final concentrations, as described in Section 4.2.15. Epidermal differentiation was achieved by keeping the cultures at the air–liquid interface for 15 days. Afterwards, hematoxylin and eosin (H&E) staining and immunofluorescence studies were carried out (see Section 4.2.15).

**Figure 10:**
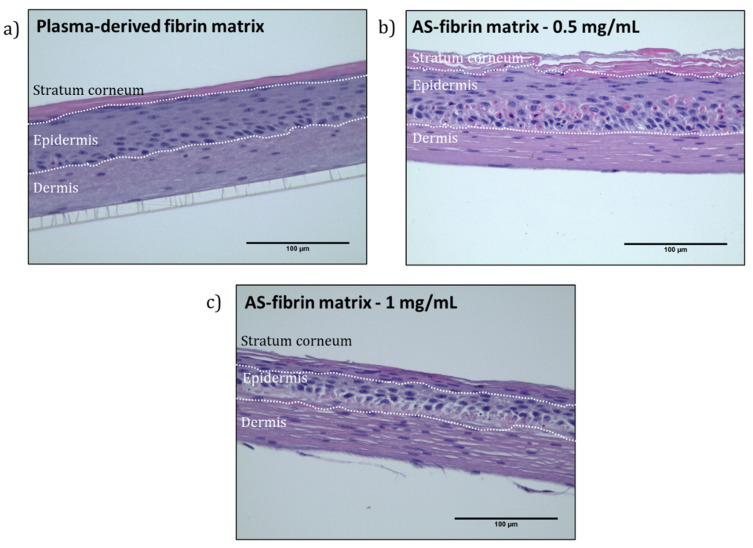
Hematoxylin and eosin (H&E) staining of (**a**) control plasma-derived fibrin dermo-epidermal skin equivalents. Skin equivalents using fibrin matrices modified with (**b**) a 0.5 mg/mL final SA concentration, and (**c**) a 1 mg/mL final SA concentration. Scale bar 100 μm.

As shown in Figure 10, H&E staining enabled the identification of the three main skin layers: the stratum corneum, epidermis, and dermis, in both the control fibrin hydrogels and the fibrin–SA matrices. Importantly, the incorporation of SA resulted in thicker and more stable structures compared to those in the control hydrogels. This improvement supports the use of longer culture periods and enhances dermo-epidermal matrix stability.

The stratum corneum, a hallmark of a mature epidermis, was visible in the control as well as in the SA–fibrin matrices. However, the presence of nuclei in the stratum corneum of matrices containing 1 mg/mL SA suggested the presence of parakeratosis, indicating incomplete terminal differentiation [57]. This could be due, at least in part, to two factors: the mechanical properties of the matrix requiring longer skin maturation periods, and the highly hydrophilic nature of sodium alginate, the water-retaining capacity of which could have prevented the complete drying necessary for terminal epidermal differentiation [58,59]. On the other hand, the matrices containing 0.5 mg/mL of SA were particularly promising for the development of well-differentiated dermo-epidermal skin equivalents.

**Figure 11:**
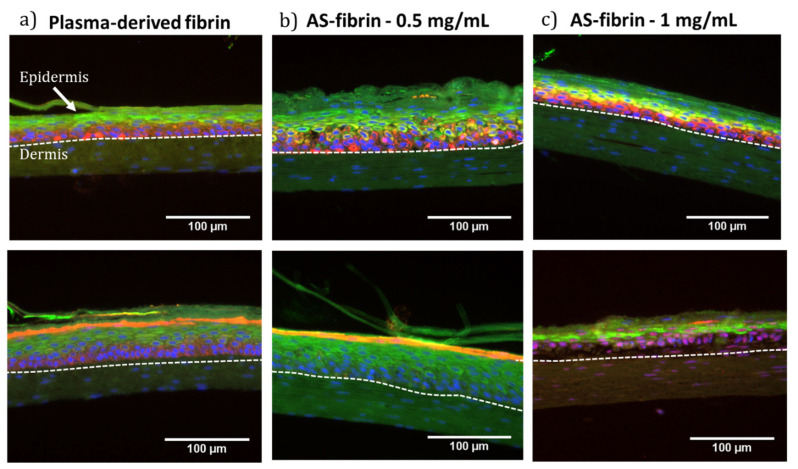
Immunostaining of (**a**) plasma-derived fibrin matrices, and SA–fibrin matrices with final concentrations of (**b**) 0.5 and (**c**) 1 mg/mL at 15 days of differentiation. The staining shows the expressions of K5 (red staining) and K10 (green staining) in the upper panels, and the expression of Involucrin (green staining) and Loricrin (red staining) in the lower panels. Cell nuclei stained with DAPI appear as blue spots. Scale bar: 100 μm.

Immunofluorescence staining analyses were also performed to study the expression of proteins widely used as markers of epidermal structure and differentiation. The proteins analyzed included cytokeratin 5 (K5), characteristic of basal cells, cytokeratin 10 (K10) and Involucrin (INV), characteristic of suprabasal cells, and Loricrin (LOR), expressed in the granular layer during the final stages of keratinocyte differentiation, with nuclei counterstained with DAPI. As seen in Figure 11, positive expression of all four markers was observed in all the samples.

K5 expression was consistent in all samples, confirming the formation of the basal layer, which contains the epidermal stem cells. K10 and INV were also similarly expressed in all samples, indicating the formation of the spinous layer. However, LOR expression revealed significant differences: while it was strongly expressed in the control and the 0.5 mg/mL SA samples, its expression was markedly reduced in the samples containing 1 mg/mL of SA (Figure 11c, lower panel), suggesting incomplete formation of the granulosum and corneum strata and thus incomplete terminal differentiation. This LOR deficit is consistent with the histological observation of parakeratosis in these samples.

The combined immunofluorescence and histological results highlight that the SA-fibrin matrices, particularly at a 0.5 mg/mL SA concentration, support the in vitro development of well-organized and differentiated dermo-epidermal skin equivalents. Meanwhile, at higher SA concentrations (1 mg/mL), despite having an overall good structure, impairments in terminal differentiation were observed, indicating the need for further optimization. The water holding capacity of SA [58,59] could be addressed by improving the drying of the culture surface and modifying the culture conditions, or adjusting the hydrogel formulation to further promote complete epidermal differentiation [45,60].

## 3. Conclusions

The main motivation behind this study was to investigate the possibility of improving the intrinsic limitations of fibrin hydrogels, particularly their mechanical stability, without compromising their favorable biological properties in supporting cell proliferation and differentiation. We conducted the in vitro generation of dermo-epidermal equivalents of human skin as a model, due to our extensive experience with this type of construct for both research and clinical applications [4,60,61,62]. The chosen approach involved the production of chemically modified fibrin hydrogels (called SA–fibrin hydrogels), using alginate modified through the incorporation of N-hydroxysuccinimide (NHS) to form succinimidyl alginate (SA). Concentrations of SA ranging from 0.5 to 3 mg/mL were tested, and these hydrogels underwent comprehensive chemical, mechanical, microstructural, and biological characterization.

At SA concentrations higher than 0.5 mg/mL (1 to 3 mg/mL), a moderate delay in hydrogel polymerization was observed, increasing from approximately 15 min to around 30 min, with higher SA concentrations compromising polymerization. Similarly, increasing SA concentration caused a gradual change in hydrogel microstructure, transitioning from a filamentous and highly porous matrix to a more fibrous and compact architecture, with reduced pore sizes. These changes in structure are form the basis of many, if not all, of the changes observed in these hydrogels. On the other hand, from a mechanical standpoint, across all SA concentrations tested, the modified hydrogels exhibited significantly enhanced properties, including the following: (1) they contracted less both in the absence (see Figure 5) and in the presence (see Figure 6) of hFBs; (2) in terms of rheological properties, they presented improved mechanical properties and viscoelastic behavior (see Figure 7).

Biological tests show that at SA concentrations up to 1 mg/mL, the hydrogels provide appropriate biocompatibility (cell viability and proliferation) for both dermal fibroblasts (see Figure 8) and epidermal keratinocytes (see Figure 9). Taken together, these data indicate that fibrin hydrogels with SA concentrations in the range of 0.5–1 mg/mL constitute an interesting new method for the production of dermo-epidermal equivalents.

From a biocompatibility perspective, SA–fibrin hydrogels with moderate to high SA concentrations (≥2 mg/mL for hFBs and ≥1 mg/mL for hKCs) showed a significant reduction in cell proliferation (see Figure 8 and Figure 9), thereby limiting the optimal SA concentration range to between 0.5 and 1 mg/mL. Finally, regarding the generation of dermo-epidermal skin equivalents, the most crucial part of this experimental approach, it was found that, using SA concentrations of 0.5 and 1 mg/mL after 15 days of culture at the liquid–air interface, both SA concentrations supported the formation of a well-structured and differentiated skin containing its key fundamental layers (a dermis and epidermis with a stratum corneum) (see Figure 10 and Figure 11). However, at the SA concentration of 1 mg/mL, H&E staining revealed the presence of cell nuclei in the stratum corneum, indicative of parakeratosis, likely resulting from incomplete epidermal differentiation. The low levels of Loricrin, a marker of terminal skin differentiation, detected via immunofluorescence, confirmed this hypothesis. This phenomenon could be attributed, at least in part, to two factors: (1) the mechanical properties of the hydrogel, which may have resulted in it requiring a longer period than the 15 days used in this study to achieve terminal epidermal differentiation, and (2) the highly hydrophilic nature of sodium alginate, the water retention capacity of which may have prevented the low degree of hydration necessary in the epidermal compartment to achieve its terminal differentiation in the cultures carried out at the liquid–air interface in these experiments. Further experiments will help to determine whether it is possible to overcome this problem and expand the range of use of these hydrogels. Meanwhile, it seems clear that using SA-functionalized fibrin hydrogels at an SA concentration of 0.5 mg/mL is a promising new method for generating dermo-epidermal equivalents for clinical, experimental or drug and cosmetic testing purposes.

## 4. Materials and Methods

### 4.1. Materials

Sodium alginate (A2158-100G, viscosity of a 2% solution at 25 °C: ~250 cps), N-hydroxysuccinimide (NHS), 1-ethyl-3-(3-dimethylaminopropyl)carbodiimide (EDC), isopropanol, NaCl, CaCl_2_ and phenylethylamine were purchased from Sigma Aldrich (Burlington, MA, USA). Amchafibrin (500 mg) was supplied by Meda Pharma SL (Madrid, Spain), Dulbecco’s modified Eagle’s medium (DMEM) was obtained from Biochrom KG, (Cambridge, UK) and fetal bovine serum (FBS) and Penicillin/Streptomycin (P/S) were purchased from Thermo Fisher Scientific (Waltham, MA, USA). The culture medium for fluorescence experiments was DMEM without phenol red (21063029) (Thermo Fisher Scientific, Waltham, MA, USA). Additionally, for hKC culture, we used DMEM including Ham-F12 and fetal calf serum (FCS) from Biochrom KG (UK); insulin, triiodothyronine, cholera toxin, adenine, hydrocortisone, and insulin from Sigma Aldrich (USA); and epithelial growth factor from Austral Biologicals (San Ramon, CA, USA). The plasma used for the experiments was freshly frozen platelet-poor human plasma (PPP) made commercially available by BioVIT (Frankfurt/Main, Germany), stored at −80 °C, using the same batch throughout the described experiments. To perform the biological tests, a Live/Dead^®^ Viability assay and AlamarBlue^TM^ Cell Viability Reagent, both from Thermo Fisher Scientific (USA), were used. For the immunofluorescence characterization of the dermo-epidermal equivalents, Cytokeratin 5 Monoclonal Antibody (PA5-32465), Cytokeratin 10 Monoclonal Antibody (MA5-13705), Loricrin Polyclonal Antibody (PA5-30583), Involucrin Monoclonal Antibody (MA5-11803), and DAPI for nuclei staining were obtained from Thermo Fisher Scientific (USA).

### 4.2. Methods

#### 4.2.1. Synthesis of Succinimidyl Alginate (SA)

An amount of 2 g of sodium alginate was dissolved in 200 mL of distilled water at room temperature. The pH of the solution was adjusted to 5 by adding HCl, and then NHS and EDC were added in a molar ratio of 1:1:3 (Alginate:EDC:NHS). After allowing the mixture to react for 2 h at room temperature through mechanical stirring, the product was precipitated in isopropanol, in which unreacted NHS and EDC products are soluble. The final product (succinimidyl alginate) was isolated by centrifugation, re-dissolved in water, and re-precipitated in isopropanol to remove most of the free EDC and NHS. Finally, the product was vacuum-dried to remove the excess isopropanol.

#### 4.2.2. Nuclear Magnetic Resonance Spectroscopy

^1^H-NMR spectra were recorded at 25 °C in 1% (*w*/*v*) solutions of both polymers in D_2_O and in Varian Gemini 300 MHz under standard conditions. The formation of the succinimide ester (NHS ester) in the alginate-repeating unit was quantified, as also certain products derived from the reaction to obtain succinimidyl-alginate. The ^1^H-NMR spectrum was first established at 80 °C and with pre-saturation by the sodium alginate before the reaction to obtain references of the alginate protons. In addition, to verify the reaction capacity of the succinimidyl ester of the alginate with primary amines, the SA was allowed to react with an excess of phenylethylamine in PBS at a ratio of NHS:NH2 of 1:3 for 1 day; it was then subsequently dialyzed for 7 days to eliminate the unreacted excess phenylethylene amine. Finally, the contents of the dialysis bag were frozen, lyophilized and analyzed by NMR. At the same time, an SA solution was dialyzed for 7 days to analyze its ^1^H-NMR spectrum.

#### 4.2.3. FTIR-ATR

Changes in chemical structure were analyzed by FTIR-ATR spectroscopy and recorded on a Spectrum One FTIR spectrometer (Perkin Elmer, Waltham, MA, USA). Succinimidyl alginate was tested in comparison with the control sodium alginate.

#### 4.2.4. Thermogravimetric Analysis (TGA)

TGA was used to analyze changes in SA and sodium alginate degradation with temperature using TA-Q500 (TA Instruments, New Castle, DE, USA). The polymers were heated from room temperature to 500 °C at a rate of 10 °C/min in a N_2_ atmosphere.

#### 4.2.5. Gel Permeation Chromatography (GPC)

The average molecular weights of the SA samples (Mw and Mn) and polydispersity were measured by gel permeation chromatography (GPC) using a Waters 1515 isocratic HPLC pump calibrated with narrow-molecular-weight-distribution pullulan standards. SA and sodium alginate were dissolved at 2 mg/mL, and an aqueous sodium nitrate solution was used as the mobile phase at a flow rate of 1 mL/min.

#### 4.2.6. Preparation of SA-Modified Plasma-Derived Fibrin Hydrogels

SA was dissolved in 0.9% *w*/*v* NaCl (NaCl solution) at different concentrations to obtain a final concentration in the hydrogel of 0.5, 1, 2 and 3 mg/mL (0.05%, 0.1%, 0.2% and 0.3% *w*/*v*, respectively). Then, the volume of the fresh platelet-poor plasma (PPP) was calculated to obtain hydrogels with a final fibrin concentration of 1.2 mg/mL (0.12% *w*/*v*); to achieve the final desired volume, an appropriated volume of NaCl solution was added. The solution was incubated at 37 °C for 15 min in a 5%—CO_2_, 70%—humidity incubator to allow for the occurrence of an amidation reaction between the succinimidyl esters present in the SA and the primary amines of the proteins, with the fibrinogen molecules present in the plasma. After 15 min, CaCl_2_ 1% (*w*/*v*) in NaCl solution was added at a concentration of 0.08% *v*/*v* to initiate fibrin polymerization, leading to the obtention of a hydrogel with a final fibrinogen concentration of 1.2 mg/mL and the aforementioned SA concentrations.

#### 4.2.7. Time and Kinetics of Gelation

To analyze the influence of SA when incorporated into the plasma solution, the gelation time of the plasma-derived fibrin/SA hydrogels was studied both by the flip-flop method and by UV spectroscopy [45]. For the flip-flop method, hydrogels (1.5 mL) were prepared in glass vials measuring 3 cm in diameter (Labbox Labware S.L, Barcelona, Spain), as described in Section 4.2.6. The vials were then tilted every 5 min. When there was no liquid left in the vial and the hydrogel remained stuck to the bottom, we considered this the gelation time. Analysis by UV spectroscopy was performed using a Synergy^TM^ HTX Multi-Mode microplate reader (Winooski, VT, USA), measuring the O.D. of 3 to 6 hydrogels (100 µL) per experimental condition in the microplate reader for 2 h, at 37 °C, at 30 s intervals and at 350 nm.

#### 4.2.8. Analysis of the Hydrogel Microstructure (SEM)

Before analyzing the hydrogels by SEM microscopy, Philips XL30 scanning electron microscope (SEM) (Eindhoven, The Netherlands), they were subjected to a supercritical point drying process. First, an exchange of water for ethanol was previously carried out to prepare samples for supercritical drying. To achieve this, the hydrogels were left in PBS for 24 h and then immersed in water/ethanol solutions until the concentration of ethanol was 100%. For the CO_2_ drying process, a Thar R100W supercritical CO_2_ reactor (Thar Technologies, Pittsburgh, PA, USA) was used at a temperature of 35 °C and a pressure of 100 bar for 90 min using a slow depressurization process (30 min). Finally, the dried hydrogels were fractured in liquid nitrogen and observed on a SEM. Average pore size and standard deviations were measured by ImageJ software version V1.54 K (by measuring 30 pores for each hydrogel.

#### 4.2.9. Rheological Properties

Rheological tests were performed on a TA Instruments AR-G2 rheometer (New Castle, DE, USA) using a sand-blasted aluminum plate (25 mm diameter). Samples were gelled one day before the experiment and incubated at 37 °C to equilibrate in PBS. A constant normal force was set for each sample at 37 °C. First, the region of linear viscoelasticity was determined by an oscillating strain sweep in which the strain values varied between 0.01 and 200% at a frequency of 0.3 Hz. To determine the relationship of G′ and G″ with frequency, a dynamic frequency sweep between 0.01 and 20 Hz for a fixed strain value obtained from the above experiment was performed.

#### 4.2.10. Contraction of Plasma-Derived Fibrin/SA Hydrogels

Hydrogels (1.5 mL) were prepared in glass vials measuring 3 cm in diameter (Labbox Labware S.L., Barcelona, Spain), as described in Section 4.2.6, left for 1 h at 37 °C in the incubator for polymerization, detached by adding 1 mL of PBS pre-warmed at 37 °C, and placed in a P60 Petri dish that had previously been weighed using a precision balance. After carefully removing the excess PBS with a pipette, the dish surface was carefully dried with cotton swabs without touching the hydrogel. After taking a picture of the hydrogels close to a scale, they were weighed in the pre-weighed Petri dish to obtain the weight of the hydrogels. Finally, they were submerged in PBS, again in the same Petri dish, and incubated at 37 °C until the next measurement. Measurements were made at 0, 1, 2, 4, 6, 8, 24, 48, and 72 h, and at 5, 7, 10, 15, 21, 25, and 30 days. The weight swelling ratio (W_t_/W_0_), which reflects the hydrogels’ shrinking at each time point, is the average of the weight of 3 to 6 hydrogels per condition at that time point (W_t_), divided by the weight of these hydrogels at time zero (W_0_).

$${SwellingRatio}_{Weight}=\frac{{Weight}_{t}}{{Weight}_{0}}$$

Areas at each time point were calculated with ImageJ image processing software using the pictures taken that were mentioned above. The areas obtained at each time point (A_t_) were divided by those obtained at time zero (A_0_) to obtain the area contraction ratio:

$${SwellingRatio}_{Area}=\frac{{Area}_{t}}{{Area}_{0}}$$

#### 4.2.11. Biological Tests and Culture of Primary Human Fibroblasts, Primary Human Keratinocytes, and Immortal Human Skin Keratinocytes (HaCaT Cells)

hFBs were obtained from biopsies of healthy donors from collections of biological samples of human origin; these samples are registered in the Spanish “National Registry of Biobanks for Biomedical Research of the Carlos III Health Institute”. In this work, cells from different donors were used, and similar results were obtained regardless of the donor. The cells were then cultured following protocols previously established by our laboratory [61,62]. Fibroblasts were cultured in DMEM containing 10% FBS and 1% P/S. Primary human keratinocytes (hKCs) (PromoCell, Heidelberg, Germany) were cultured in accordance with the procedures of Rheinwatd, J. G., and Green et al., who used irradiated mouse embryonic fibroblasts (3T3-J2) used to create the feeder layer necessary for keratinocyte growth [63]. The culture medium used comprised DMEM and Ham-F12 in a 3:1 ratio, enhanced with 10% FCS, 5 μg/mL of insulin, 1.3 ng/mL of triiodothyronine, 8 ng/mL of cholera toxin, 24 μg/mL of adenine, 0.4 μg/mL of hydrocortisone, and 5 μg/mL of insulin, along with 1% P/S and 10 ng/mL of EGF (Epidermal Growth Factor). Since hKCs need the feeder layer, in the Live/Dead (Dead (see Section 4.2.13) and AlamarBlue (see Section 4.2.14) assays, immortal human skin keratinocytes (HaCaT cells; PromoCell, Germany) were used. HaCaT cells were cultured under the same conditions as hFBs were. The fluorescence experiments on hKCs and HaCaT, such as the Live/Dead assay or AlamarBlue assay, used the same DMEN but without phenol red.

#### 4.2.12. Area Contraction of Plasma-Derived Fibrin/SA Hydrogels with hFBs

Briefly, 1.5 mL hydrogels containing hFBs at a concentration of 20,000 cells/mL were produced in glass vials, as described in Section 4.2.10.

The cells were added before gel polymerization, after 15 min of reaction between SA and plasma. Next, the antifibrinolytic agent Amchafibrin was added at a final concentration of 0.008% (*w*/*v*), and lastly, CaCl_2_ was added at a final concentration of 0.08% (*v*/*v*) to start the coagulation cascade leading to the polymerization of plasma fibrinogen into fibrin. The hydrogels were incubated for 1 h at 37 °C to achieve complete polymerization, and then detached from the vials by adding 3 mL of pre-warmed DMEM at 37 °C, placed in Petri dishes and photographed close to a scale bar. This time point was considered time zero. The hydrogels were immersed in DMEM, which was renewed every 2 days. As described in Section 4.2.10, at each time point, the hydrogels were photographed, their area was calculated with ImageJ image processing software, and the areas at each point (A_t_) were used to calculate the contraction ratio kinetics with respect to the area at time zero (A_0_). Noteworthy is that the hydrogels did not adhere to the Petri dish; they were suspended in the culture media. The time points were as follows: 0, 1, 2, 4, 6, and 8 h, and 1, 2, 3, 5, 7, and 10 days.

#### 4.2.13. Live/Dead Fluorescence Assays

To assess cell viability, the Live/Dead assay was performed using the Live/Dead^®^ Viability/Cytotoxicity Kit for mammalian cells (Thermo Fisher Scientific, USA). This kit differentially stains live cells with green-fluorescent Calcein AM (CaAM) and dead cells with red-fluorescent ethidium homodimer-1 (EthH) based on membrane integrity [64]. The hydrogels (200 µL) were first polymerized directly within μ-Slide 8-well glass-bottom plates (Ibidi GmbH, Gräfelfing, Germany; 0.34 cm^2^ per well). For the hFB assay (human primary fibroblasts), cells were added to the hydrogel mixture at a final concentration of 160,000 cells/mL prior to polymerization. Then, polymerization was induced by calcium addition (CaCl_2_). After hydrogel polymerization, the cells remained embedded within the hydrogel matrix. For HaCaT cells, 5000 cells were seeded on top of previously polymerized hydrogels (approximately 14,700 cells/cm^2^). Cell viability was assessed at days 3 and 7 of culture. The hydrogels were washed three times with PBS for 5 min to remove residual media. Then, 100 µL of staining solution consisting of 0.5 µL/mL CaAM and 1 µL/mL EthH in PBS was added to each hydrogel. Samples were incubated at 37 °C for 40 min, protected from light. After incubation, the staining solution was removed and replaced with 100 µL of pre-warmed PBS at 37 °C. Samples were imaged using a Leica-SPE confocal microscope (Leica, Wetzlar, Germany). For hFBs, z-stack images were acquired and maximum-intensity projections were generated. The emission wavelength of EthH can be visualized at 570–625 nm with an excitation wavelength of 561 nm, and the emission wavelength of CaAM can be visualized at 500–555 nm with an excitation wavelength of 488 nm. For each experimental time point (days 3 and 7) and for each cell type (hFBs or HaCaT keratinocytes), each hydrogel with the corresponding SA concentration, along with the plasma-derived fibrin control hydrogel, was analyzed. Three hydrogels were evaluated for each condition.

#### 4.2.14. AlamarBlue Assay

The AlamarBlue assays involved the preparation of plasma-derived fibrin hydrogels without and with the corresponding SA concentrations and cells (hFBs and HaCaTs). They were performed at 1, 3, and 7 days of culture. The hydrogels were polymerized in 96-well plates. Each well included 100 µL of SA–plasma hydrogel containing embedded 800 hFB cells, or surface-seeded 3400 HaCaT cells. At each time point, the hydrogels in the wells were washed 3 times with 100 µL of PBS. Then, 100 µL of a 10% (*v*/*v*) AlamarBlue^TM^ reactive dissolved in PBS was added following the supplier’s instructions. Subsequently, the hydrogels were incubated for 3 h at 37 °C, and the supernatants’ fluorescence was measured at 600 nm using Synergy^TM^ HTX Multi-Mode Microplate Reader (Winooski, VT, USA). Six hydrogels were evaluated for each condition.

#### 4.2.15. Dermo-Epidermal Skin Constructs. H&E Staining and Immunostaining Assays

The characterization of the SA–plasma hydrogels concluded by analyzing their suitability as matrices with which to generate 3D organotypic skin equivalents with primary hKCs and hFBs. Dermo-epidermal skin constructs were generated in transwells (Fisher Scientific, Frederick, MA, USA), as described in Montero, A. et al. [45], and in Section 4.2.6, using 4 mL/transwell SA–plasma hydrogels containing a 0.5 or 1 mg/mL final concentration of SA. The dermal compartment was generated by adding 20,000 hFBs/mL of hydrogel along with CaCl_2_, and incubating the constructs for 2 h at 37 °C in a cell culture incubator (SCO_2_W) (Shel Lab CO_2_, Cascade Technical Sciences, Inc., Hillsboro, OR, USA). Afterwards, the hydrogels were covered with 5 mL of fibroblast culture medium and further incubated for 24 h at 37 °C, with 5% CO_2_ and at 40% relative humidity. Subsequently, 3 mL of keratinocyte culture media was used to seed 1 million hKCs on top of the plasma-derived fibrin hydrogels (240.000 cells/cm^2^), in order to generate the epidermal compartment. The cells were then allowed to adhere to the hydrogel surface at 37 °C in a cell culture incubator for 48 h. Under these conditions, the adhered keratinocytes formed a confluent layer. Transwells were transferred onto Falcon^TM^ 6-well deep-well TC-treated polystyrene plates (Thermo Fisher Scientific, USA) to achieve epidermal differentiation at the air–liquid interface. Then, the culture medium for hKCs described in Section 4.2.11 was modified to contain 0.5% FCS and a 50 µg/mL ascorbic acid, and enough medium was added to the transwells to cover the dermis but leave the epidermis in contact with the air. The dermo-epidermal hydrogels were then incubated for 15 days, changing the culture medium every two days. Afterwards, the skin equivalents were subjected to histological and immunohistochemical analysis. Dermo-epidermal equivalents were first histologically characterized by hematoxylin and eosin (H&E) staining, in accordance with the protocol used by Fischer A. et al. [65]. For further characterization, immunofluorescence studies were performed using antibodies against Involucrin, Loricrin, Cytokeratin 5 and Cytokeratin 10 (described in paragraph 1. Materials), and the preparations were stained for 5 min with DAPI to visualize the nuclei of the cells in blue fluorescence. A Leica Dmi8 inverted microscope (Leica, Wetzlar, Germany) was used to examine and take pictures of the samples.

## Figures and Tables

**Table 1:** Assayed reaction parameters used to obtain succinimidyl alginate and attain the chosen modification degrees. We used an NHS:EDC molar proportion of 3:1 with respect to alginate.

Precipitation Solvent	Reaction Time (min)	% NHS
Ethanol	30	14.8
Ethanol	60	13.0
Dioxane	30	11.1
DMF	30	13.6
Isopropanol	30	13.0
Isopropanol	120	26.8

**Table 2:** Flip-flop analysis of the SA included in plasma-derived hydrogels. The hydrogels’ gelation time is displayed in minutes. N = 3.

SA Final Concentration in Hydrogel (mg/ML)	Gelification Time (Every 5 Min)	Gelification (Yes/No)
Control (0 mg/mL)	15	Yes
0.5	10	Yes
1.0	10	Yes
1.5	15	Yes
2.0	15	Yes
2.5	15	Yes
3.0	15	Yes
3.5	20	Yes
4.0	20	Yes

## Data Availability

The original contributions presented in this study are included in the article. Further inquiries can be directed to the corresponding authors.

## Abbreviations

The following abbreviations are used in this manuscript:

SA	Succinimidyl alginate
NHS	N-hydroxysuccinimide
H&E	Hematoxylin and eosin
EDC	Carbodiimide
hFBs	Human fibroblast
hKCs	Keratinocye

## References

[1] Fan L., Cao M., Gao S., Wang T., Wu H., Peng M., Zhou X., Nie M. (2013). Preparation and characterization of sodium alginate modified with collagen peptides. Carbohydr. Polym..

[2] Zhang X., Wan H., Lan W., Miao F., Qin M., Wei Y., Hu Y., Liang Z., Huang D. (2022). Fabrication of adhesive hydrogels based on poly (acrylic acid) and modified hyaluronic acid. J. Mech. Behav. Biomed. Mater..

[3] Strehin I., Ambrose W.M.I., Schein O., Salahuddin A., Elisseeff J. (2009). Synthesis and characterization of a chondroitin sulfate-polyethylene glycol corneal adhesive. J. Cataract Refract. Surg..

[4] Sanz-Horta R., Matesanz A., Gallardo A., Reinecke H., Jorcano J.L., Acedo P., Velasco D., Elvira C. (2023). Technological advances in fibrin for tissue engineering. J. Tissue Eng..

[5] Yang J., Steck J., Suo Z. (2020). Gelation kinetics of alginate chains through covalent bonds. Extrem. Mech. Lett..

[6] Lee J.M., Edwards H.H.L., Pereira C.A., Samii S.I. (1996). Crosslinking of tissue-derived biomaterials in 1-ethyl-3-(3-dimethylaminopropyl)-carbodiimide (EDC). J. Mater. Sci. Mater. Med..

[7] Bax D.V., Nair M., Weiss A.S., Farndale R.W., Best S.M., Cameron R.E. (2021). Tailoring the biofunctionality of collagen biomaterials via tropoelastin incorporation and EDC-crosslinking. Acta Biomater..

[8] Yang Y., Yang Y. (2016). Chapter 5—Side Reactions Upon Amino Acid/Peptide Carboxyl Activation. Side Reactions in Peptide Synthesis.

[9] Adamiak K., Sionkowska A. (2020). Current methods of collagen cross-linking: Review. Int. J. Biol. Macromol..

[10] Golunova A., Velychkivska N., Mikšovská Z., Chochola V., Jaroš J., Hampl A., Pop-Georgievski O., Proks V. (2021). Direct and Indirect Biomimetic Peptide Modification of Alginate: Efficiency, Side Reactions, and Cell Response. Int. J. Mol. Sci..

[11] Kim Y.S., Guo J.L., Lam J., Grande-Allen K.J., Engel P.S., Mikos A.G. (2019). Synthesis of Injectable, Thermally Responsive, Chondroitin Sulfate-Cross-Linked Poly(N-isopropylacrylamide) Hydrogels. ACS Biomater. Sci. Eng..

[12] Wang X., Li Y., Li Q., Neufeld C.I., Pouli D., Sun S., Yang L., Deng P., Wang M., Georgakoudi I. (2017). Hyaluronic acid modification of RNase A and its intracellular delivery using lipid-like nanoparticles. J. Control. Release Off. J. Control. Release Soc..

[13] Chan A.T., Karakas M.F., Vakrou S., Afzal J., Rittenbach A., Lin X., Wahl R.L., Pomper M.G., Steenbergen C.J., Tsui B.M.W. (2015). Hyaluronic acid-serum hydrogels rapidly restore metabolism of encapsulated stem cells and promote engraftment. Biomaterials.

[14] Khadka R., Carraher C., Hamiaux C., Travas-Sejdic J., Kralicek A. (2020). Synergistic improvement in the performance of insect odorant receptor based biosensors in the presence of Orco. Biosens. Bioelectron..

[15] Strehin I., Nahas Z., Arora K., Nguyen T., Elisseeff J. (2010). A versatile pH sensitive chondroitin sulfate–PEG tissue adhesive and hydrogel. Biomaterials.

[16] Chang C.Y., Chan A.T., Armstrong P.A., Luo H.-C., Higuchi T., Strehin I.A., Vakrou S., Lin X., Brown S.N., O’Rourke B. (2012). Hyaluronic acid-human blood hydrogels for stem cell transplantation. Biomaterials.

[17] Zhang X., Ma Z., Ke Y., Xia Y., Xu X., Liu J., Gong Y., Shi Q., Yin J. (2021). An injectable serotonin–chondroitin sulfate hydrogel for bio-inspired hemostatic adhesives with high wound healing capability. Mater. Adv..

[18] Vig K., Chaudhari A., Tripathi S., Dixit S., Sahu R., Pillai S., Shree R.S. (2017). Advances in skin regeneration using tissue engineering. Int. J. Mol. Sci..

[19] Jeong K.H., Park D., Lee Y.C. (2017). Polymer-Based Hydrogel Scaffolds for Skin Tissue Engineering Applications: A Mini-Review. J. Polym. Res..

[20] Stojic M., López V., Montero A., Quílez C., de Aranda I.G., Vojtova L., Velasco D. (2019). Skin Tissue Engineering. Biomaterials for Skin Repair and Regeneration.

[21] Choi J., Kim H., Choi J., Oh S.M., Park J., Park K. (2014). Skin corrosion and irritation test of sunscreen nanoparticles using reconstructed 3D human skin model. Environ. Health Toxicol..

[22] Reddy M.S., Ponnamma D., Choudhary R., Sadasivuni K.K. (2021). A comparative review of natural and synthetic biopolymer composite scaffolds. Polymers.

[23] Llames S.G., Del Rio M., Larcher F., Garcia EGarcia MEscamez M.J., Jorcano J.L., Holguin P., Meana A. (2004). Human plasma as a dermal scaffold for the generation of a completely autologous bioengineered skin. Transplantation.

[24] Kuo J.W., Swann D.A., Prestwich G.D. (1991). Chemical modification of hyaluronic acid by carbodiimides. Bioconj. Chem..

[25] McDonagh B.H. (2012). Optimalised Carbodiimide Chemistry for RGD-Coupled Alginate. Master’s Thesis.

[26] Li M., Shi X., Yang B., Qin J., Han X., Peng W., He Y., Mao H., Kong D., Gu Z. (2022). Single-component hyaluronic acid hydrogel adhesive based on phenylboronic ester bonds for hemostasis and wound closure. Carbohydr. Polym..

[27] Iwasawa T., Wash P., Gibson C., Rebek J. (2007). Reaction of an Introverted Carboxylic Acid with Carbodiimide. Tetrahedron.

[28] Saeger M. (2016). Effect of Carbodimide Functionalization Chemistry on Alginate Structure and Hydrogel Properties. Master’s Thesis.

[29] Schulz A., Gepp M.M., Stracke F., von Briesen H., Neubauer J.C., Zimmermann H. (2019). Tyramine-conjugated alginate hydrogels as a platform for bioactive scaffolds. J. Biomed. Mater. Res. Part A.

[30] Kamimura W., Hattori R., Koyama H., Miyata T., Takato T. (2012). A calcium-cross-linked hydrogel based on alginate-modified atelocollagen functions as a scaffold material. J. Biomater. Sci. Polym. Ed..

[31] Zhang M., Yang J., Deng F., Guo C., Yang Q., Wu H., Ni Y., Huang L., Chen L., Ding C. (2019). Dual-functionalized hyaluronic acid as a facile modifier to prepare polyanionic collagen. Carbohydr. Polym..

[32] Hua J., Li Z., Xia W., Yang N., Gong J., Zhang J., Qiao C. (2016). Preparation and properties of EDC/NHS mediated crosslinking poly (gamma-glutamic acid)/epsilon-polylysine hydrogels. Mater. Sci. Eng. C Mater. Biol. Appl..

[33] Fernandes-Cunha G.M., Chen K.M., Chen F., Le P., Han J.H., Mahajan L.A., Lee H.J., Na K.S., Myung D. (2020). In situ-forming collagen hydrogel crosslinked via multi-functional PEG as a matrix therapy for corneal defects. Sci. Rep..

[34] Shpichka A.I., Koroleva A.V., Deiwick A., Timashev P.S., Semenova E.F., Moiseeva IYa Konoplyannikov M.A., Chichkov B.N. (2017). Evaluation of the vasculogenic potential of hydrogels based on modified fibrin. Cell Tissue Biol..

[35] Shpichka A.I., Konarev P.V., Efremov Y.M., Kryukova A.E., Aksenova N.A., Kotova S.L., Frolova A.A., Kosheleva N.V., Zhigalina O.M., Yusupov V.I. (2020). Digging deeper: Structural background of PEGylated fibrin gels in cell migration and lumenogenesis. RSC Adv..

[36] Wang S., Zhao Q., Li J., Du X. (2022). Morphing-to-Adhesion Polysaccharide Hydrogel for Adaptive Biointerfaces. ACS Appl. Mater. Interfaces.

[37] Dadashzadeh A., Moghassemi S., Amorim C.A. (2021). Evaluation of PEGylated fibrin as a three-dimensional biodegradable scaffold for ovarian tissue engineering. Mater. Today Chem..

[38] Jiang L., Dong X., Chen L., Han R., Hao P., Wang L., Gao J., Chen X., Li X. (2023). A composite hydrogel membrane with shape and water retention for corneal tissue engineering. Heliyon.

[39] Kavitha N., Karunya T.P., Kanchana S., Mohan K., Sivaramakrishnan R., Uthra S., Kapilan K., Yuvaraj D., Arumugam M. (2019). Formulation of alginate based hydrogel from brown seaweed, Turbinaria conoides for biomedical applications. Heliyon.

[40] Hu C., Lu W., Mata A., Nishinari K., Fang Y. (2021). Ions-induced gelation of alginate: Mechanisms and applications. Int. J. Biol. Macromol..

[41] Vorwald C.E., Gonzalez-Fernandez T., Joshee S., Sikorski P., Leach J.K. (2020). Tunable Fibrin-Alginate Interpenetrating Network Hydrogels to Support Cell Spreading and Network Formation. Acta Biomater..

[42] Sanz-Horta R., Matesanz A., Jorcano J.L., Velasco D., Acedo P., Gallardo A., Reinecke H., Elvira C. (2022). Preparation and Characterization of Plasma-Derived Fibrin Hydrogels Modified by Alginate di-Aldehyde. Int. J. Mol. Sci..

[43] Natesan S., Stone R., Coronado R.E., Wrice N.L., Kowalczewski A.C., Zamora D.O., Christy R.J. (2019). PEGylated Platelet-Free Blood Plasma-Based Hydrogels for Full-Thickness Wound Regeneration. Adv. Wound Care.

[44] Montalbetti C., Falque V. (2005). Amide Bond Formation and Peptide Coupling. Tetrahedon.

[45] Montero A., Acosta S., Hernández R., Elvira C., Jorcano J.L., Velasco D. (2021). Contraction of fibrin-derived matrices and its implications for in vitro human skin bioengineering. J. Biomed. Mater. Res. Part A.

[46] Lee F., Kurisawa M. (2013). Formation and stability of interpenetrating polymer network hydrogels consisting of fibrin and hyaluronic acid for tissue engineering. Acta Biomater..

[47] Burmeister D.M., Roy D.C., Becerra S.C., Natesan S., Christy R.J. (2018). In Situ Delivery of Fibrin-Based Hydrogels Prevents Contraction and Reduces Inflammation. J. Burn Care Res. Off. Publ. Am. Burn Assoc..

[48] Caliari S.R., Burdick J.A. (2016). A practical guide to hydrogels for cell culture. Nat. Methods.

[49] Murphy K.C., Whitehead J., Zhou D., Ho S.S., Leach J.K. (2017). Engineering fibrin hydrogels to promote the wound healing potential of mesenchymal stem cell spheroids. Acta Biomater..

[50] Somasekharan L., Kasoju N., Raju R., Bhatt A. (2020). Formulation and Characterization of Alginate Dialdehyde, Gelatin, and Platelet-Rich Plasma-Based Bioink for Bioprinting Applications. Bioengineering.

[51] Sarker B., Singh R., Silva R., Roether J.A., Kaschta J., Detsch R., Schubert D.W., Cicha I., Boccaccini A.R. (2014). Evaluation of Fibroblasts Adhesion and Proliferation on Alginate-Gelatin Crosslinked Hydrogel. PLoS ONE.

[52] Li S., Nih L.R., Bachman H., Fei P., Li Y., Nam E., Dimatteo R., Carmichael S.T., Barker T.H., Segura T. (2017). Hydrogels with precisely controlled integrin activation dictate vascular patterning and permeability. Nat. Mater..

[53] Liu J.C., Tirrell D.A. (2008). Cell Response to RGD Density in Cross-Linked Artificial Extracellular Matrix Protein Films. Biomacromolecules.

[54] Tan J.J.Y., Nguyen D.-V., Common J.E., Wu C., Ho P.C.L., Kang L. (2021). Investigating PEGDA and GelMA Microgel Models for Sustained 3D Heterotypic Dermal Papilla and Keratinocyte Co-Cultures. Int. J. Mol. Sci..

[55] Chen X., Liu C., McDaniel G., Zeng O., Ali J., Zhou Y., Wang X., Driscoll T., Zeng C., Li Y. (2024). Viscoelasticity of Hyaluronic Acid Hydrogels Regulates Human Pluripotent Stem Cell-derived Spinal Cord Organoid Patterning and Vascularization. Adv. Healthc. Mater..

[56] Kong D., Nguyen K.D.Q., Megone W., Peng L., Gautrot J.E. (2017). The culture of HaCaT cells on liquid substrates is mediated by a mechanically strong liquid–liquid interface. Faraday Discuss.

[57] Song J., Shea C.R. (2010). Benign versus malignant parakeratosis: A nuclear morphometry study. Mod. Pathol..

[58] Berger F.M., Ludwig B.J., Wielich K.H. (1953). The hydrophilic and acid binding properties of alginates. Am. J. Dig. Dis..

[59] Savić Gajić I.M., Savić I.M., Svirčev Z. (2023). Preparation and Characterization of Alginate Hydrogels with High Water-Retaining Capacity. Polymers.

[60] Montero A., Quílez C., Valencia L., Girón P., Jorcano J.L., Velasco D. (2021). Effect of Fibrin Concentration on the In Vitro Production of Dermo-Epidermal Equivalents. Int. J. Mol. Sci..

[61] Meana A., Iglesias J., Del Rio M., Larcher F., Madrigal B., Fresno M.F., Martin C., San Roman F., Tevar F. (1998). Large surface of cultured human epithelium obtained on a dermal matrix based on live fibroblast-containing fibrin gels. Burn J. Int. Soc. Burn Inj..

[62] Del Rio M., Larcher F., Serrano F., Meana A., Muñoz M., Garcia M., Muñoz E., Martin C., Bernad A., Jorcano J.L. (2002). A preclinical model for the analysis of genetically modified human skin in vivo. Hum. Gene Ther..

[63] Rheinwald J.G., Green H. (1975). Serial cultivation of strains of human epidermal keratinocytes: The formation of keratinizing colonies from single cells. Cell.

[64] Gantenbein-Ritter B., Sprecher C.M., Chan S., Illien-Jünger S., Grad S. (2011). Confocal imaging protocols for live/dead staining in three-dimensional carriers. Methods Mol. Biol..

[65] Fischer A.H., Jacobson K.A., Rose J., Zeller R. (2008). Hematoxylin and eosin staining of tissue and cell sections. CSH Protoc..

